# Progress in Gene Therapy for Hereditary Tyrosinemia Type 1

**DOI:** 10.3390/pharmaceutics17030387

**Published:** 2025-03-18

**Authors:** Helen Thomas, Robert C. Carlisle

**Affiliations:** 1 Department for Continuing Education, University of Oxford, Headington, Oxford OX1 3PJ, UK; helen_cooper2000@yahoo.co.uk; 2 Institute of Biomedical Engineering, Department of Engineering Science, University of Oxford, Headington, Oxford OX3 7DL, UK

**Keywords:** tyrosinemia, gene therapy, lentivirus, LNP

## Abstract

Hereditary Tyrosinemia Type-1 (HT1), an inherited error of metabolism caused by a mutation in the fumarylacetoacetate hydrolase gene, is associated with liver disease, severe morbidity, and early mortality. The use of NTBC (2-(2-nitro-4-fluoromethylbenzoyl)-1,3-cyclohexanedione) has almost eradicated the acute HT1 symptoms and childhood mortality. However, patient outcomes remain unsatisfactory due to the neurocognitive effects of NTBC and the requirement for a strict low-protein diet. Gene therapy (GT) offers a potential single-dose cure for HT1, and there is now abundant preclinical data showing how a range of vector-nucleotide payload combinations could be used with curative intent, rather than continued reliance on amelioration. Unfortunately, there have been no HT1-directed clinical trials reported, and so it is unclear which promising pre-clinical approach has the greatest chance of successful translation. Here, to fill this knowledge gap, available HT1 preclinical data and available clinical trial data pertaining to liver-directed GT for other diseases are reviewed. The aim is to establish which vector-payload combination has the most potential as a one-dose HT1 cure. Analysis provides a strong case for progressing lentiviral-based approaches into clinical trials. However, other vector-payload combinations may be more scientifically and commercially viable, but these options require additional investigation.

## 1. Introduction

### 1.1. Hereditary Tyrosinemia Type 1

HT1 is a rare, mendelian inherited, autosomal recessive, inborn error of tyrosine metabolism caused by a deficiency of the enzyme fumarylacetoacetate hydrolase (FAH). Worldwide prevalence of HT1 is around 1 in 100,000 births, but significant geographical variations exist, with one area of Quebec having a frequency of 1 in 1800 births and many countries where no known mutations exist due to lack of diagnosis [1].

**Figure 1:**
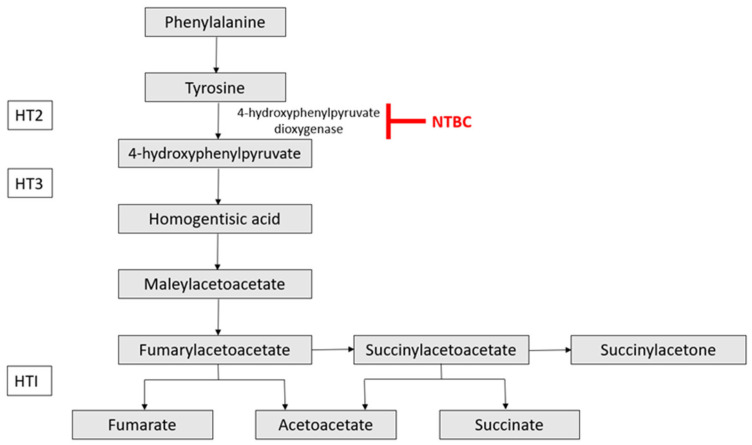
Tyrosine metabolism pathway, including Hereditary Tyrosinemia Type 1, 2, and 3 associated with different steps within the pathway and the point at which NTBC interference occurs.

FAH is the last of five enzymes within the tyrosine catabolic pathway, and pathogenic mutations within the *FAH* gene result in a defective final metabolism step, causing an accumulation of toxic metabolites fumarylacetoacetate (FAA), maleylacetoacetate (MAA), and succinylacetone (SA) [2]; see Figure 1. The build-up of toxins causes cellular oxidative stress leading to liver disease, renal tubulopathy, and porphyria-like syndrome and can lead to hepatocellular carcinoma (HCC) [3]. HT1 is classified as either acute, subacute, or chronic, but there are no mild phenotypes that do not require treatment. Acute patients present before the age of 2 months with acute liver failure; subacute patients present between 2 and 6 months of age with liver disease, and those with the chronic form present later with liver cirrhosis and rickets [4]. If left untreated, patients suffer from acute episodes of metabolic crisis and death from liver failure, HCC, or porphyria crisis, usually before the age of 2 [5]. North America and many European countries implement a neonatal screening program for HT1 to start treatment immediately and prevent liver and other organ damage [6].

### 1.2. Current Treatment and Its Limitations

In 1992, a clinical trial investigating NTBC (generic name Nitisinone) to treat five HT1 patients showed remarkable results [7]. NTBC is a potent inhibitor of 4-hydroxyphenyl pyruvate dioxygenase (HPPD)—the second enzyme in the tyrosine catabolism pathway (see Figure 1). NTBC blocks the tyrosine metabolism pathway prior to the FAH step and thereby prevents accumulation of the toxic metabolites responsible for hepatic and renal symptoms. However, it has a concomitant side effect of accumulation of tyrosine; therefore, HT1 patients must also adhere to a strictly regulated diet [3]. NTBC and diet control have radically changed the long-term prognosis of HT1 patients by vastly reducing morbidity and mortality, with most patients experiencing normal growth. However, long-term treatment adherence is a substantial issue [5], with associated reduced quality of life outcomes [8] as well as increased healthcare costs due to higher numbers of hospitalisations [3]. In addition, the risk of HCC and neuropsychological and neurocognitive issues remains high [9]. Indeed, one analysis showed that low phenylalanine in the first year of life and high tyrosine thereafter, caused by NTBC use in combination with lack of adherence to diet, correlates with behavioral problems [10].

Despite the limitations of NTBC described above, the supportive healthcare economics case for its use versus no treatment has been made by Simoncelli et al. [11]. However, NTBC and its associated regimen of dietary supplements and monitoring are expensive, with annual costs approaching £1.7 M by our calculations.

In summary, NTBC has unquestionably changed the lives of patients with HT1, allowing better quality of life and freedom from severe, acute manifestations, but as with many other inherited disorders, treatment does not address the underlying mutation causing the disease. Several monogenic diseases with high treatment costs and unsatisfactory outcomes or QoL are now experiencing fresh investment in clinical development of GT in pursuit of a one-shot cure [12]. The high cost of HT1 treatment and management, coupled with the obvious adherence and neurocognitive outcome issues, provides justification to explore a clinical development strategy to find a GT cure for HT1.

### 1.3. GT as a Potential Cure for HT1

#### 1.3.1. HT1 Genetics

The *FAH* gene is located on chromosome 15, spanning 30–35 kb and including 14 exons. The cDNA has an open reading frame of 1257 base pairs encoding 419 amino acids [13]. Nearly 100 pathogenic (predominantly missense) mutations are recorded that cause HT1, with large clusters in exons 9 and 12 where substrate binding domains exist, but no clear genotype-phenotype map has been established to date [14]. Patients carry either homozygous or heterozygous mutations, and disease-causing alleles are found in varying prevalence throughout the world, with certain high-frequency alleles in Quebec and northern Europe [13].

#### 1.3.2. Technologies for Liver Directed GT

The liver acts as the bioreactor for the production and secretion of an abundance of life-sustaining proteins, including FAH. It is the ideal environment for recombinant protein production and therefore the target of many GT approaches for various diseases, including Haemophilia A and B [15]. Furthermore, the liver has huge regenerative potential [16], and studies in *FAH^−/−^* mice have demonstrated that FAH+ cells have a strong selective proliferation advantage, allowing healthy repopulation without 100% initial transfer of WT gene copy [17]. Notably, several vectors with natural or engineered tropism for, and high levels of infection of, the liver have been identified [18].

Leading the way in liver-directed GT vectors are adeno-associated virus (AAV) vectors, which have demonstrated significant, dose-dependent hepatic infection and positive results in the clinic [19,20]. Their simple structure and lack of pathogenic viral genes mean they are less immunogenic than other viral vectors but at present are not suitable for patients with existing immunity to specific serotypes. AAVs have limited coding capacity and, following entry into the nucleus, predominantly exist episomally, so their concentration and thus transgene expression reduce over time due to cell turnover [21]. Approval by the FDA of liver-directed AAV GTs, Roctavian, and Hemgenix for Haemophilia A and B, respectively, and the use of AAV as delivery vehicles for insertional or editing genetic material currently in clinical trials demonstrates their utility and potential (NCT04581785 and NCT05222178).

Lentiviruses (LVs) have potential as vectors for liver-directed GT but have demonstrated lower transduction efficiency and are prone to pro-inflammatory responses [19]. LVs have been successful in several ex vivo applications, such as for Gaucher and Fabry disease [22], and can transfect both dividing and non-dividing cells, leading to durable transgene expression [23].

LNPs may be another attractive vector from a safety perspective but require liver trophic optimisation to allow non-toxic doses capable of delivering nucleic acid material to the nucleus of hepatocytes [24] as current success, e.g., Patisiran, is based on cytoplasmic acting nucleotide payloads [25].

Indeed, as well as vector choice and delivery mechanism, the nucleotide payload is of paramount importance in defining the potential efficacy of a GT system. Thanks to the application of CRISPR Cas9 and other DNA splicing proteins such as transposons and zinc finger nucleases, preclinical data illustrating potential options are abundant. GT systems have commonly depended on the delivery of cDNA encoding the full or partial transgene and have often relied on episomal maintenance (most AAV systems) or integration into the genome (LVs) with the advantage of stable, durable expression and inheritance by daughter cells, but with the risk of insertional mutagenesis. Substantial research has resulted in improved safety of LVs, including the creation of self-inactivating LV vectors with lower insertional mutagenesis risk and disrupted promoter/enhancer activity [26].

CRISPR Cas9 and its derivatives, such as Cas9 nickase (Cas9n), offer a plethora of options for liver-directed GT tools [27]. They create precise DNA strand breaks (double (DSB) or single), providing an opportunity for transgene integration via homology-directed repair (HDR) mechanisms [28]. HDR does not work efficiently in post-mitotic cells like healthy hepatocytes; therefore, non-homologous end joining (NHEJ) is the more common DNA repair mechanism used. This seals the DSB without homology, resulting in random insertions and deletions, known as indels, which in turn lead to random gene-edited products that may or may not be functional but could pose a safety risk due to unpredictability [29]. Mechanisms and environments that encourage HDR over NHEJ lead to higher frequencies of precise genome changes and more predictable outcomes. The neonatal liver and hepatocytes under liver-injury stress represent such environments where gene expression profiles favour proliferation and HDR, resulting in more efficient editing and expansion of the correction [28]. Single-strand breaks caused by Cas9n encourage HDR and prevent NHEJ and can be used in adult hepatocytes for precise genome editing, although efficiency is low [30].

Precise gene editing using next-generation CRISPR-Cas technology such as base editors and prime editors or by providing small homology-repair templates can also be employed to edit specific mutations that are already in use in clinical trials [31]. As well as low HDR editing efficiency, other issues with CRISPR products include immunogenicity against these proteins of bacterial origin and the generation of off-target indels, an issue that can be ameliorated by encoding Cas9 at the RNA level, which has a shorter expression duration. The research and development of CRISPR products is currently advancing at such a pace that strategies to overcome limitations are rapidly being realised [32].

The potential therapeutic power of CRISPR-Cas technology is being realised in early-phase clinical trials where it has demonstrated the ability to knockout specific genes within human cells to produce disease-alleviating or curative results from a single IV administration of liver trophic LNPs [33,34,35].

The number of different GT systems being explored in preclinical and clinical development at present is at an all-time high, with several potential systems that could address HT1.

#### 1.3.3. The Patient

HT1 presents in infancy and is identified in many countries during neonatal screening, and to gain maximum benefit from GT, dosing would ideally occur in neonates. This would also reduce the risk of liver damage that can occur as the neonatal liver is able to grow and regenerate quickly. However, clinical trials are more likely to start in adult HT1 patients who may have liver disease despite NTBC treatment. This is important to consider because the effectiveness of GT may be affected by the environment within the diseased liver [36].

This study will review the vectors and payloads available to guide the progression of HT1 GT to the clinic. The desire to identify curative GT treatments requiring single or minimal dosing means this review will exclude cell-based and RNA or oligonucleotide-based therapies as well as ex vivo approaches and the extra cost and complexity they involve.

## 2. Materials and Methods

A comprehensive literature search was performed using the electronic databases PubMed [37] and, as a validation step, Ovid-Embase [38] to retrieve English language papers. Publications reporting pre-clinical studies with results investigating the use of GT systems in animal models of Hereditary Tyrosinemia Type 1 were of interest. These were identified using the criteria and process, and their potential biases are described in Appendix A.

## 3. Results

### 3.1. Pre-Clincal Results

#### 3.1.1. Analysis Summary

Forty publications describing 39 HT1 preclinical model studies were identified. A variety of different vectors and payload combinations were investigated as shown in Appendix A. Five studies aimed to perform a knockout of the 4-hydroxyphenylpyruvate dioxygenase (*HPD*) gene rather than correct the mutation in the *FAH* gene, resulting in an animal with the more benign HT3 phenotype instead of correcting the HT1 phenotype.

It is noteworthy that 31 of 39 studies rely on the *FAH^−/−^* mouse model. This model shows strong selective proliferation of corrected hepatocytes, and as the field of research relies on it so heavily, discussion of it is warranted. As the *FAH^−/−^* mouse model is frequently used in the field of liver-directed GT studies, most studies analysed were proof of concept studies. Many were less than 2 months in duration, potentially not long enough to see their full potential for repopulation, or they did not include sufficient analysis for conclusions to be drawn. Maintaining *FAH^−/−^* animals on NTBC has the same effect as in humans, in that it maintains LFTs and kidney function at near-normal levels and prevents liver and kidney damage. Withdrawing NTBC induces *FAH^−/−^* liver cell damage due to the near-immediate production and accumulation of toxic metabolites, and this cell injury causes prolific cell turnover, thus inducing an environment where corrected cells can have a selective advantage.

#### 3.1.2. Controls

All studies used at least one control. WT (*FAH^+/+^*) and *FAH^−/−^* animals maintained on NTBC, used as positive controls, always survived and grew steadily and had normal liver and kidney biochemical marker levels. As negative controls, *FAH^−/−^* mice receiving no NTBC were dosed with no treatment or phosphate buffered saline, or even an empty or partial GT vector. Such controls typically lost weight and died within about 4 weeks of NTBC withdrawal or were euthanised if they lost >20% of their body weight. Their liver and kidney function tests as well as tyrosine, SA, and AFP levels were supraphysiological, and histological analysis confirmed liver disease.

#### 3.1.3. Vector Free Systems

Twelve vector free studies were identified [39,40,41,42,43,44,45,46,47,48,49,50]. Their application method (which is typically dependent on hydrodynamic delivery) would be limited in a clinical environment, but there are still learnings to be taken regarding the utility of the payload chosen.

##### Transposons, Integrase and Viral Sequences

Early work with transposons has not been followed up since 2012 [39,40,41,42]. Similarly, work using φC31 integrase has not led to further reports, perhaps due to concerns over integration in the untranslated region of the *Cdkn1a (p21)* gene, a transcriptional target of the tumour suppressor p53 [43]. A study by Eggenhoffer et al. revealed that AAV sequences could be included in plasmid DNA to increase transgene expression longevity, but expansion was not particularly high (50%), and no integrational analysis data were included [44].

##### Plasmids Expressing Cas9,sgRNAs, and Repair Template

More recently, studies have investigated the injection of plasmids expressing Cas9, gRNAs, and repair templates with homology arms. The first corrected a single nucleotide polymorphism (SNP) via HDR [45], and the second used two gRNAs to correct the same mutation but by splicing and replacing exon 8 via microhomology-mediated end joining (MMEJ), an innate mechanism that occurs more frequently in non-dividing cells than HDR and may, therefore, be more efficient [46]. Yin et al. demonstrated that optimisation of the sgRNA had a significant effect on outcomes with up to 0.4% initial editing efficiency expanding to 33.5% FAH+ hepatocytes at day 30. The on-target indel rate was ~26% and off-target < 0.3% with no off-target cleavage sites detected. It was acknowledged that naked injection of Cas9 systems is unlikely to be applicable clinically, and efficient delivery vectors would be needed [45]. Although the MMEJ study reported a high initial editing efficiency of 5.18% ± 1.92%, which increased to 64.42% FAH+ hepatocytes 30 days after NTBC withdrawal, in contrast, FAH mRNA levels remained comparatively low at 13.45% compared to normal. This short, proof-of-concept study could warrant further investigation using delivery vectors [46].

##### Oligonucleotides Expressing *Hpd* Knockout Cas9 and sgRNAs

Another study demonstrating the conversion of HT1 to HT3 double mutants (*Fah^−/−^/Hpd^−/−^)* were performed via knockout of the *Hpd* gene using naked injection of Cas9 plus gRNAs designed to excise *Hpd* gene exons, resulting in average editing efficiencies of 8% and 92% at 1 and 8 weeks, respectively, measured via *Hpd^−/−^* proliferation. Biochemistry levels were comparable to HT1 mice maintained on NTBC for 8 weeks [47]. Gu et al. also demonstrated *Hpd* knockout using single-cell *FAH^−/−^* pig embryos, which were micoinjected with Cas9 mRNA and sgRNA into the cytoplasm. The resulting double mutants exhibited the more benign phenotype of HT3 and were protected against lethal liver injury. No hepatocellular injury or body weight loss was observed at 2 years, and liver function and inflammatory cytokines were comparable to WT [48]. These studies highlight that favourable results can be achieved via knockout, but crucially this is not a cure for HT1.

##### Prime Editors

Two recent studies demonstrated *Fah* gene editing using the relatively new CRISPR-Cas9 next generation of prime editors (PE). Both achieved low initial editing efficiency (0.76% and 0.07% FAH+) and expansion by day 40 [49,50]. The on-target indel rate was low at 0.4–1.2%, and off-target analysis of 15 sites revealed no indels [50].

Whilst these studies of non-vectorised approaches have limited translational potential due to their reliance on the injection of very high volumes at high pressures over short time frames (in mice and rats, injection volumes sometimes exceeded total blood volume), they have been of value in providing a comparison of the integration efficiency and safety of non-viral approaches and so have helped identify technologies that may be optimally encoded within a virus or complexed within a non-viral system.

#### 3.1.4. Lipid Nano Particles

##### Adenine Base Editor (ABE) Plus sgRNA

Recently, a codon-optimised adenine base editor (ABE), RA6.3 plus sgRNA, was delivered first as plasmids using hydrodynamic injection and then packaged as mRNA within an LNP and injected into the tail vein of mice maintained on NTBC for 6 days before withdrawal and assessment. Unpackaged cargo had a far greater initial editing efficiency of 9.5% ± 4% compared to the LNP delivery, which achieved 0.44% ± 0.28%, suggesting LNP may need further optimisation to harness the high potential editing efficiency of the ABE. The duration of this study did not allow assessment of liver repopulation [51].

A multi-dose study of LNP delivery of the same ABE at 1 mg/kg and sgRNA at 0.5 mg/kg, both given four times, three days apart, followed [52]. Higher initial editing efficiency at the target site was achieved at 12.5% ± 2.67%, and widespread FAH+ patches and weight gain were observed by day 58. This was the highest reported initial editing achieved of any HT1 GT animal study; however, these total dose levels in humans would be much higher than those used in ongoing clinical trials using LNP delivery as described in Section 3.2.4 and could be intolerable and/or toxic. Interestingly, bystander indel rates were reduced to 0.096% compared to 1.9% caused by plasmid delivery in the previous study, suggesting the short half-life of LNP-delivered ABE mRNA might minimise off-target effects in vivo [52].

#### 3.1.5. Retroviral Vector Studies

Two GT preclinical studies were carried out in *FAH^−/−^* mice with mutations in exon 5 [53,54], retroviral (RV) vectors encoding a human *FAH* transgene and a viral SV40 promoter. The first study utilised a single injection of the vector and transgene directly into the portal vein 7 days after discontinuing NTBC or 2 days after partial hepatectomy, both of which induced liver injury with the aim of causing cell regeneration and proliferation. Only 29 of 56 mice survived to 2 months post-injection, and by 3–5 months post-injection, 27 of them had mosaic livers with nodules of FAH excreting (FAH+) cells and areas showing hepatocellular dysplasia and necroinflammation. Only two mice had normal liver histology with FAH+ nodules expressing 2–37% of WT FAH levels with an average of at least one copy of RV DNA per cell [53].

Five injections of the RV vector via an intra-portal catheter between day 2 and 11 following partial hepatectomy or NTBC withdrawal were then tested [54]. At 48 h post-dosing, staining by IHC showed an average of <1% cells to be FAH+. All mice grew normally despite the absence of NTBC, and no macroscopic disease histology was visible at 8–12 weeks post-treatment. FAH enzyme levels were 20–80% (average 59%) of WT levels, and an average of 1 RV DNA copy per hepatocyte was found throughout the liver, and >80% of the liver was FAH+ by 12 weeks, demonstrating the selective proliferative advantage of corrected cells. AST (aspartate aminotransferase), bilirubin, and SA measurements were reduced to normal levels by 12 weeks. This therefore represents an impressive advance in helping demonstrate the potential of this approach. In combination with its precursor, useful information on dosing, levels of integration required, and levels of enzyme expression needed was obtained. However, all livers had at least 5–10% *FAH^−/−^* cells remaining at 12 weeks, which could potentially be a risk for HCC [55]. As the study was relatively short (12 weeks), no HCC assessment could be made. The study could have been strengthened by gene integration site and anti-vector and transgene immunological assessment, as well as renal function analysis [53].

In 1998, Overturf et al. published another RV study that used an ex vivo GT process on the same mouse model. This is not described in detail here due to the ex vivo approach being outside the remit, but amino acid levels were partially normalised compared to WT mice, and FAH+ cell repopulation was shown to be patchier in the livers of RV-*FAH^+/+^* transplanted mice than those transplanted with WT hepatocytes [54].

#### 3.1.6. Lentiviral Vectors

##### Ex Vivo *FAH* Gene Cassettes Plus Promoters

Extensive research using HT1 pig models, supported by mouse toxicology, with either in vivo or ex vivo delivery of lentiviral (LV) vector systems containing a full *FAH* gene cargo with liver-specific promoters, has been performed. These 5 studies (6 publications) represent the most detailed and reproducible preclinical HT1 GT data available, and the findings are strengthened by reliance on a large animal model of HT1 and the long-term data (almost 3 years) available. Findings are summarised in Table 1.

The technical complexity and consequent cost, time, and regulatory issues raised by ex vivo approaches and the progression to in vivo studies mean further detailed analysis of this work is not reported here. However, the highly impressive levels and durations of expression, corrective liver function, and health achieved are notable guides to what may be achievable if in vivo delivery efficiency can be optimised. This ex vivo success was exemplified by one pig, which was euthanised at 988 days after birth due to the inability to house such a large experimental animal. LFTs, tyrosine and SA levels, AFP (HCC biomarker), and histological examination revealed no divergence from WT, and a complete liver repopulation with LV-FAH+ hepatocytes was seen [56].

##### In Vivo Delivery of *FAH* Gene Cassettes Plus Promoter

To progress from ex vivo studies, a full therapeutic dose toxicology study was performed using the LV-FAH vector administered intravenously via the tail vein of WT *FAH^+/+^* mice [57]. Induction of chemical liver injury via *N*-nitrosodiethylamine/carbon tetrachloride (DC) was used to assess specific toxicity in fibrotic liver tissue where modified gene expression may exist. Mice receiving LV ± coadministration of DC had 1–2% transduced hepatocytes at the end of the study (day 106), without a proliferation advantage, and all animals gained weight, although mice in the LV+DC group gained slightly less weight. The LV-FAH-only group had normal LFTs and AFP measurements. DC-induced liver injury parameters were apparent and were exacerbated by the addition of LV-FAH, resulting in high LFTs, AFP, and slightly higher liver and spleen-to-body weight ratios. No evidence of tumorigenicity in any group was observed in this study [57].

Following this, Nicolas et al. intravenously dosed HT-1 pigs with LV-FAH [58]. Ear vein injection caused an anaphylactic reaction with acute hypotension, despite the use of prophylactic immunosuppression, the absence of pre-exposure, and regardless of the gene encoded. Hence, it was determined to be driven by complement responses to the LV vector. Ultrasound-guided, percutaneous portal vein infusion circumvented this, and four HT1 pigs were successfully dosed with LV-FAH. NTBC was discontinued after infusion and cycled on and off until weight stabilised. One pig was euthanised after 60 days and 3 cycles of NTBC, at which point evenly distributed liver FAH+ cell expansion had reached ~10%. The other two pigs achieved NTBC independence at days 78 and 98. At day 142, all tests, including tyrosine measurements, were normalised to WT levels. At 225 days post-treatment, liver biopsies displayed 69% and 78% FAH+ cells, but also multiple abnormalities and fibrosis patches remained. By the end of the study (day 337), FAH+ cells had almost completely repopulated the liver, and the fibrosis and inflammation had reduced to WT levels. Cure of the in vivo LV-FH pigs was faster than those treated by the ex vivo system [36], providing further support for the in vivo approach. In complete contrast, the control *FAH^−/−^* pig undertreated on NTBC developed severe liver damage, high AFP, and HCC by 12 months [58].

PCR on multiple tissues at days 2, 60, and 337 showed integration was restricted to the liver. This was not the case for the pig injected via the ear vein, where LV-FAH had integrated into the cells of many organs. Hepatic integration site analysis revealed higher exonic integration at day 60 but more intronic integration by day 377, and a trend for non-tumour-associated gene integration by day 337 was also seen. Gene expression profiling via RNA sequencing was performed, revealing LV-FAH-treated pigs had similar profiles to NTBC-sustained HT1 pigs at 6 months but were most like WT pig expression profiles by 12 months post-treatment [58].

This study represents a step change in providing compelling efficacy and safety data from a long-term study in a large animal model, and although portal vein dosing is not ideal, it has favourable clinical translatability versus ex vivo approaches. It would not be surprising to see this technology enter clinical testing in the coming years. With more probing of the optimal design and dose of LV, the heterogeneity of NTBC dependence and FAH positivity may be reduced. Addressing the need for ultrasound-guided percutaneous portal vein infusion by reducing complement and enhancing liver tropism will be important for future clinical ease of use. Table 2 summarises in vivo vectored systems data.

#### 3.1.7. Adenovirus Vectors

##### *FAH* Gene Plus Promoter Cassette

The first HT1 Adenovirus (AdV) vector study encoded the human *FAH* gene driven by the RSV LTR promoter (FAHAdRSV) and was delivered by tail or portal vein injection into mice [59]. Results served to emphasise the unsuitability of the short-term expression provided by standard non-integrating AdVs.

##### Base Editor (BE) and Cas9 Approaches

Two studies used Ad-based vectors to deliver base editing and Cas9 approaches [30,60]. Whilst pre-clinical outputs were strong, it is felt the high level of off-target infection, pre-existing immunity, immunogenicity, and pathogenicity of Ad will preclude its clinical development in this context, so this work will not be discussed further here.

#### 3.1.8. AAV Vectors

##### AV Vector Containing cDNA of *FAH* Gene Under Control of Either CMV or Albumin Promoter

Chen et al. attempted to correct mouse *FAH^−/−^* hepatocytes using an AAV vector (serotype not overtly stated) transporting cDNA of the *FAH* gene under liver-specific promoter control. All AAV-FAH treated animals overcame initial weight loss after NTBC was withdrawn at 4 weeks and survived. At 5 months, FAH+ expressing nodules constituted 50–90% of the liver. The vector was present at <0.1 copies/cell in a subset of mice, which could be increased to 0.5 copies/cell at 5–8 months after NTBC withdrawal if NTBC was given until 3 months post-treatment, once again demonstrating the selective advantage for repopulation. SA in the urine remained elevated in treated mice after 5 months, and ALT levels reduced but not to WT levels, suggesting partial phenotype rescue. Tyrosine and total bilirubin levels were reduced to WT levels. Non-malignant hepatoma was observed in 5/7 mice after 9 months—something that has not been reported in other studies. It is likely that a subpopulation of *FAH* genes integrated into the genome and expanded, which is indirectly supported by evidence of separate integration events showing homogenous staining within nodules but variations between nodules; however, no integration analysis was performed [61].

##### AAV2 or AAV8 Containing a 4.5 kb Homologous Repair Template of the *FAH* Gene

Paulk et al. described partial amelioration of HT1 using either an AAV2 or AAV8 vector expressing a 4.5 kb genomic DNA fragment homologous to murine Fah flanked by 2.25 kb homology arms administered intravenously to *FAH^−/−^* neonatal or adult mice at different doses. Minor differences between AAV8 and AAV2 were seen, but regardless of vector, dose, or life stage, liver function could not match that achieved with NTBC [62].

##### AAV2 or AAV8 with FAH Expression Cassette and Ribosomal DNA Homology Arms

The Paulk 2010 study provided a homology repair template but no additional guide for integration specificity. Another short-term integration study investigated if the introduction of ribosomal DNA (rDNA) homology repeats into the payload would increase integration frequency, especially into rDNA repeats by HDR or NHEJ. AAV-rDNA-hFah mice stabilised weight immediately, whereas the control mice receiving AAV with stuffer DNA, not rDNA, required 4 cycles of NTBC. AAV8 produced higher copy numbers per cell than AAV2. Concerningly, integrations were observed in different organs throughout the body, including the heart, muscle, lung, and kidney, as well as the liver [63]. Higher site-directed integration was achieved using the rDNA homology arms but was widespread and non-specific.

##### AAV2 or AAV8 Dual Vectors, One Containing Cas9 and the Other sgRNA Plus FAH DNA as Homologous Recombination Donor

Studies in previous section used one AAV and repair templates with/without homology arms to encourage HDR; in a subsequent study, SpCas9 and a sgRNA were encoded in two separate AAVs to investigate whether editing efficiency could be increased [29]. This size of cargo requires dual AAV vectors. No promoter was used, so only homologous recombination events could lead to gene correction and restore *FAH* gene expression. Neonatal or adult mice were dosed via co-injection into the facial vein with either both vectors or just the AAV-sgRNA-HDR vector and followed for 28 days with NTBC given throughout to discourage selective growth. Neonates maintained on NTBC grew normally without any sign of hepatocyte toxicity. After 28 days, FAH+ hepatocytes inhabited 10.8% of the liver in mice receiving both AAVs, whereas controls without AAV-Cas9 displayed very few FAH+ nodules. Adult mice received vectors at a higher dose (4 × 10^11^ vg/mouse of AAV-sgRNA-repair and 2 × 10^11^ vg/mouse of AAV-Cas9), but those receiving both AAVs demonstrated editing efficiency of 1.6% vs. negligible levels in single AAV controls. A small cohort was then followed for an additional 4 weeks after NTBC withdrawal; neonates showed significant growth increase and correction to 80% FAH+, which was much higher than those receiving only AAV-sgRNA-HDR, which lost weight but did exhibit FAH+ expansion [29]. This study shows the dependency on Cas9 for much greater editing efficiency, the power of the growing neonatal liver for faster repopulation, and demonstrates a potentially safer method for withdrawing NTBC after more significant expansion. It would be interesting to perform the same study again with Cas9n instead of WT Cas9. No off-target activity analysis was performed.

Another study used a similar two AAV8 vector system with similar cargoes [64]. The main difference was the use of *FAH^−/−^* Rabbits which have much larger livers than mice. This model has a 10 bp deletion in exon 2 of the *FAH* gene. A 15-day-old rabbits, maintained on NTBC, were co-administered two AAVs or PBS via ear vein injection, and NTBC was withdrawn 5 days later. All AAV-treated rabbits survived, gained weight, and exhibited normalised LFTs and liver and kidney histology similar to age-matched WT rabbits at 5 and 9 months old, with widespread FAH+ cells observed throughout the liver. Deep sequencing revealed HDR-mediated correction frequency between 0.9 and 3.71% at different sites within the livers. On-target, NHEJ-mediated indels of between −38 bp and +68 bp were identified at frequencies between 5.31 and 9.15%, but it was noted that the in-frame insertions or deletions of amino acids caused by these indels may not have any functional effect on the FAH protein and may instead contribute to the therapeutic effect of the AAV-Cas9 system. Although the HT1 phenotype was rescued in these rabbits, the editing efficiency was still low. Subsequently, neonatal rabbits were injected with the same system at 7 days old and euthanised 7 days later when HDR-mediated gene correction levels were between 1.71 and 4.13% (i.e., higher than the levels observed after 5 months in the 15-day-old treated rabbits). Off-target effects of Cas9 were investigated, and levels were comparable with WT animals. Detailed RNA sequencing analysis was also performed and showed that the AAV-treated HT1 rabbits had global gene expression profiles in the liver similar to WT rabbits, including near-normal expression of genes within the tyrosine metabolism pathway. Immune response genes were also analysed, and again the AAV-treated animals had similar expression patterns to WT animals except for ILR10, suggesting a mild immune response to AAV8. Untreated HT1 rabbits had a completely different gene expression profile. One of the AAV-treated rabbits went on to mate and produce healthy heterozygous offspring. This study suggests correction via this AAV-Cas9 system is safe and well-tolerated [64].

Mondal et al. used a similar dual AAVDJ system to deliver spCas9, gRNAs, and a homologous repair template to correct an SNP in exon 8 of the *FAH* gene. This time, treatment was conducted in utero via direct co-injection into the foetal liver at day 15 of gestation. Six mice were euthanised at day 0, 3, or 4 after weaning, and FAH+ 2–4 cell nodules were found scattered throughout the liver. Of the four-day 58 mice, only one required a single cycle of NTBC to rescue weight, and all others gained weight without NTBC. These three mice were euthanised at around 2 months, and the mouse that had received the higher dose (2 × 10^10^ compared to 1 × 10^10^) had significantly higher on-target allele repair of 25.1% compared to <4%. All three day 58 mice had LFTs and tyrosine levels as per WT mice. FAH+ staining at days 4, 28, and 49 post-weaning increased from 5% to 15% and then >90%, respectively, with no histological abnormalities. Onsite indels caused by NHEJ were present at a frequency of between 6 and 20%, but the polyploid nature of hepatocytes means some (>30%) will have HDR repaired alleles as well. Minimal (~0.75%) off-target mutations were reported in treated mice compared to WT mice at <0.5%. The study also investigated optimal gene correction timing via HDR and found that 14-day-old neonatal hepatocytes possessed the highest amount of DNA repair gene expression, coinciding with the most efficient genomic correction window [28]. This impressive data suggests that in utero delivery may be an efficacious and well-tolerated therapy that would take advantage of high HDR repair mechanisms of growing livers, but it is unclear whether it has advantages or greater risks over neonatal dosing. It would certainly add technical complexity to clinical deployment.

##### Dual AAV8 Vectors, One Containing SaCas9 Plus sgRNA Targeting the Apoa1 3′ UTR and the Other Including Apoa1 Homologous Sequence and the Human *FAH* Transgene

This study investigated targeted insertion of the *FAH* transgene to enable permanent expression of the transgene under the control of the *Apoa1* gene promoter [65]. Similar studies have been performed using other specific loci, including ongoing GT PKU and MMA clinical trials without Cas9 (Section 3.2.6). The dual AAV8 system, including the AAV-saCas9-gRNA and AAV-Donor (Apoa1 sequence homology arms and *FAH* transgene), was co-administered via intraperitoneal injection, and NTBC was withdrawn 7 days later, then followed until day 40. AAV-treated mice showed a transient decrease in body weight around day 20, then fully recovered. Integration analysis showed high HDR-modified *Apoa1* alleles versus NHEJ insertion via PCR comparison of the HDR (886 bp) insertion rather than the NHEJ (1810 bp) insertion of AAV-Donor at the Apoa1 cut site. The HDR-corrected hepatocytes exhibited clonal expansion more frequently than hepatocytes with NHEJ insertions. FAH protein levels were restored to 50% of WT levels, and LFTs were significantly reduced but not to WT levels. AAV-treated mice showed unaffected levels of plasma apoA1, despite the high rate of Apoa1 targeting and expansion [65].

Yang et al. [66] investigated different sgRNAs-BE4max combinations and doses to edit SNPs in the stop codon of the *FAH* gene. Two AAV8 vectors packaging the split cargo BE were injected via the tail vein. NTBC was withdrawn 7 days later in the first experiment and after 21 days in the following experiment. Editing efficiency and FAH+ cell expression were measured at the time of NTBC withdrawal and 2 months later. This study reported high initial editing efficiencies using sgRNA equalling 3.8% of allele reads at NTBC withdrawal (day 7). FAH+ cells were not observed at this time but did expand by 2 months. Finally, high-dose sgRNA with mice maintained on NTBC for 21 days resulted in an editing efficiency ~3-fold higher than the 7 days on NTBC group with many FAH+ cells at withdrawal, which expanded for a further 2 months. LFTs were almost at WT levels at day 67, and FAH mRNA was about 60% of WT for the high-dose sgRNA group but lower in other groups. Bystander and indel mutations were found elsewhere, and these varied in frequency depending on which sgRNA was used [66]. This study shows some promising results, especially for initial editing efficiency, and highlights the importance of sgRNA optimisation to maximise editing efficiency.

##### Dual AAV8 System Packaging a Split Prime Editor

Liu et al. described the two AAV8 vector system packaging a split prime editor (PE) delivered to adult mice via tail vein injection with the aim of correcting a single nucleotide G to A mutation. The main emphasis of the study was the design of the PE and phenotypic rescue data were brief. At day 24 post-treatment and NTBC withdrawal, 1.3% of alleles had a corrected point mutation, and FAH+ cells were observed [67].

The manufacturing and dosing complexities created by the need to deliver two AAV vectors have driven a move to incorporate all required components within a single AAV.

##### All-in-One AAV8 Expressing Cas9 Orthologs Plus a sgRNA for *Hpd* Knockout

These studies describe the knockout of the *Hpd* gene to ameliorate the HT1 phenotype by creating a double mutant *Fah^−/−^/Hpd^−/−^* using a compact Cas9 ortholog plus a sgRNA packaged into a single AAV vector. Neisseria meningitidis Cas9 (NmeCas9) was used, and AAV-sgRNA-hNmeCas9 was delivered via tail vein injection to adult mice with NTBC withdrawal at day 7. All AAV-sgRNA-hNmeCas9-treated mice achieved indels with frequencies in the range of 35–60% after 43 days, likely due to the expansion of edited cells. Observed liver damage was substantially less severe than in untreated *FAH^−/−^* mice [68].

A study using Streptococcus thermophilus CRISPR1-Cas9 (St1Cas9) injected into neonatal mice also successfully knocked out the *Hpd* gene and ameliorated the HT1 phenotype illustrated by reduced urine SA levels and stabilised growth. While NTBC was maintained, editing efficiencies of between 22 and 40% were achieved, again highlighting the power of the growing neonatal liver, and increased to 22–55% four months after NTBC withdrawal as measured by indel rates found in total liver DNA. These measurements are probably underestimated due to hepatocytes being ~70% of total liver mass [69].

##### All-in-One AAV8 Vector Nme2Cas9 Plus sgRNA Plus a 358 bp HDR Donor Sequence Including PAM

Ibraheim et al. continued to develop the all-in-one AAV8 vector system and compared IV-injected cleaved (self-inactivating) and uncleaved vectors in adult mice with the intention of identifying if cleaved vectors have potential safety advantages by reducing NmeCas9 expression over time [70]. NGS identified that both cleaved and uncleaved vectors enabled HDR-mediated transduction in about 0.1% of cells initially, and FAH+ nodules appeared in the liver during the 5 weeks on NTBC. Expansion post-NTBC withdrawal reached 4.4% and 4.7% with the uncleaved and cleaved at 6 weeks. NHEJ vs. HDR editing, LFT normalisation, and FAH mRNA levels were similar between the two groups. Nme2Cas9 and sgRNA levels decreased over time in the cleaved cohort, suggesting that the cleaved vector can partially reduce unwanted long-term expression of Nm2Cas9 but not significantly. No off-target editing was detected [70].

**Table 1:** Preclinical Lentiviral GT study data summary.

Year	Author	Cargo	Animal	In Vivo/ Ex Vivo	Administration	Initial FAH+ Cell Estimate and Timepoint After NTBC Withdrawal	Later FAH+ Cell Estimate and Timepoint After NTBC Withdrawal	Biochemical Normalisation Achieved	Other Information
2022	Nicolas et al. [58]	FAH gene and promoter	Pigs	In-vivo	Percutaneous ultrasound guided portal vein	10% 2 months	75% 7 months almost 100% 12 months	Normalised by 142-Days	Normal growth after NTBC cycles, normal histology at D337
2020	Nicolas et al. [36]	FAH gene and promoter	Pigs	Ex-vivo	Transplantation via injection into multiple mesenteric lymph nodes	No data	67–100% 8 months	Normalised to WT levels at 8 months	Normal growth after NTBC cycles, liver damage in *Fah*^−^ regions
2019	Kaiser et al. [57]	FAH gene and promoter	*FAH^+/+^* Mice	In-vivo	Tail vein injection	No data	1–2% D106 w/out selection	DC induced liver injury parameters were slightly exacerbated by addition of LV-FAH	Tox study in *FAH^+/+^* mice
2019	Hickey et al. [56]	FAH gene and promoter	Pigs	Ex-vivo	Transplantation of hepatocytes via portal vein infusion	No data	Almost 100% 1 year	Normalised to WT levels at 1 year and remained normal for follow up 3 years	Normal growth after NTBC cycles. 1 pig followed for 3 years. Complete amelioration and no tumours and able to reproduce.

**Table 2:** Pre-clinical study data for vectored in vivo GT systems.

Year	Author	Vector	Cargo	Animal	Administration	Initial FAH+ Cell Estimate and Timepoint After NTBC Withdrawal	Later FAH+ Cell Estimate and Timepoint After NTBC Withdrawal	Allele Correction and Timepoint After NTBC Withdrawal	Average FAH Levels Compared to WT and Timepoint After NTBC Withdrawal	Biochemical Normalisation	Other Information
**Adeno-associated Viral Vectors**											
2000	Chen et al. [61]	1 AAV	FAH gene and promoter	Mice	Intrasplenic or direct liver lobe injection	No data	50–90% 5 month	<0.1 per cell D0 0.5 per cell 3 months	No data	Partial rescue by 5 months. SA and ALT remained slightly high	Weight rescued. Non malignant hepatoma devloped.
2012	Wang et al. [63]	1 AAV2 or 8	FAH gene and ribosomal DNA homology arms	Mice	Tail vein injection	No data	0.092% 3 weeks	No data	No data	No data	Normal growth after NTBC cycles
2010	Paulk et al. [62]	1 AAV2 or 8	Repair template with homology arms	Mice	Facial vein injection	No data	50% 11 weeks	0.1% 3weeks	No data	Partial improvement at 12 weeks	Weight rescued
2021	Ibraheim et al. [70]	1 rAAV	Cas9, gRNA and repair template	Mice	Tail vein injection	0.1% D0 (after 5 weeks on NTBC)	4.7% 6 weeks	No data	No data	Normal LFTs by 6 weeks	Weight rescued
2022	Liu et al. [67]	2xAAV8	Prime Editor	Mice	Tail vein injection	No data	No data	1.3% D24	No data	No data	Weight rescued
2021	De Giorgi et al. [65]	2xAAV8	Cas9, gRNAs, FAH transgene + Apoa1 homology	Mice	Intraperitoneal injection	No data	No data	No data	50% D40	Reductions	Weight rescued
2021	Li et al. [64]	2xAAV8	Cas9, gRNA and repair template	Rabbits	Neonatal ear vein injection	No data	Widespread 5 months and 9 months	1.71–4.13% HDR D7 Neonates	No data	Adults had normal LFTs and histology at 11 weeks	Weight rescued
2022	Mondal et al. [28]	2xAAV-DJ	Cas9, gRNA and repair template	Mice	In-utero direct fetal intrahepatic injections	5% D4	15% D28 >90% D49	25.1% 2 month	No data	Normalised to WT levels at 2M	Normal growth
2021	Zhang et al. [29]	2xrAAV2/8	Cas9, gRNA and repair template	Mice	Neonatal facial vein injection	10.8% in neonates 1.6% adults D0 (after 28 days on NTBC)	80% neonates 4W	No data	No data	No data	Normal growth
2020	Yang et al. [66]	2xrAAV8	Base Editor	Mice	Tail vein injection	No data	No data	3.8% D0 after 7 days NTBC >11% D0 after 21 days NTBC	60% D67	Almost normal at D67	Weight rescued
**Adenoviral Vectors**											
2018	Shao et al. [30]	2 AdVs	Cas9, gRNA and repair template	Rat	Tail vein injection	0.1% D0	60% 3 months 95% 9 months	No data	No data	Normalised to WT levels at 3 months	Fibrosis remianed at 3M but none at 9M
1997	Overturf et al. [59]	AdV	FAH gene and promoter	Mice	Tail or portal vein injection	No data	43% 2–9 months	No data	2.4–39% Peaked at 2 months	Partial rescue 2–9 months	Partial weight rescue. 9/13 HCC development. Renal disease persisted
**Lipid Nanoparticle Vectors**											
2020	Jiang et al. [52]	LNP	Base Editor	Mice	Tail vein injection	No data	Widespread patches D58	12.5% D0 after 12D NTBC and 4 doses	Majority correctly spliced D58	No data	Weight rescued
2020	Song et al. [51]	LNP	Base Editor	Mice	Tail vein injection	No data	No data	0.44% D0 after 6D NTBC	No data	No data	No data
**Lentiviral Vectors**											
2022	Nicolas et al. [58]	LV	FAH gene and promoter	Pigs	Percutaneous ultrasound guided portal vein	10% 2 months	75% 7 months almost 100% 12 months	N/A	No data	Normalised by 142D	Normal growth after NTBC cycles, normal histology at D337
2019	Kaiser et al. [57]	LV	FAH gene and promoter	*FAH^+/+^* Mice	Tail vein injection	No data	1–2% D106 without selection	N/A	No data	DC induced liver injury parameters were exacerbated by addition of LV-FAH	Tox study in *FAH^+/+^* mice
**Retroviral Vectors**											
1996	Overturf et al. [53]	RV	FAH gene and promoter	Mice	5 injections via an intra-portal catheter	<1% 2D	>80% 12 months	1 copy per cell 12 week	59% 12 weeks	Normalised at 12 weeks	Normal growth. HCC development in first experiment
**Combined Vectors**											
2016	Yin et al. [71]	AAV and LNP	Cas9, gRNA and repair template	Mice	Intravenous injection	6.2% D0	Widespread D30	0.81% D0	9.5% mRNA D0	Normalised to WT levels at 1 month	Normal growth by D30

#### 3.1.9. Combined Vector Approaches

##### LNP Encapsulating mRNA Cas9 (4.5 kb) Plus an AAV Delivering an sgRNA and DNA Repair Template (AAV-HDR)

A series of preparatory studies were performed to investigate delivery kinetics using a viral and non-viral vector combination and to explore differences in expression and off-target activity between transient mRNA nano-Cas9 and long-term viral Cas9 [71]. Cas9 mRNA/LNP, injected intravenously into mice, was expressed in the liver at 4 and 14 h post-injection, but levels markedly diminished by 24 h, whereas sgRNA, delivered via AAV, expression was detected by day 3 but had 10× higher expression at day 7 and 14. *FAH^−/−^* mice, with a SNP, were intravenously injected with AAV-HDR, and 7 days later, LNP-mRNA Cas9 was injected to ensure coordinated maximal expression of components. NTBC was withdrawn at day 14, and some mice were sacrificed to explore the initial correction without expansion, which reached an impressive dose-dependent maximum of 6.2% FAH+ stained hepatocytes and 9.5% FAH mRNA expression compared to WT. Deep sequencing confirmed nucleotide correction in 0.81% of total liver DNA, which, if 60% of the liver is made up of hepatocytes and a factor of 4 is used to account for hepatocyte ploidy, equates to 5.4%, close to the 6.2% FAH+ staining estimation. On-target indel rates of 24.1% were reported, probably due to higher NHEJ activity compared to HDR. Less than 0.3% off-target indels were observed, which was comparable with control mice that received AAV-HDR only, suggesting the transient expression of Cas9 achieved the goal of avoiding off-target activity. At D30 after NTBC withdrawal, all mice demonstrated normal growth. LFTs were reduced to WT levels, and widespread FAH+ patches were evident. This impressive data showed a high % of initial FAH+ corrected cells and expansion resulting in the rescue of the WT HT1 phenotype as illustrated by survival without NTBC cycling and LFTs at WT levels by day 30 [71]. No quantitative measurements were available for the day 30 cell repopulation data, and the high dose of LNP-Cas9 might be prohibitive in humans. Whilst the approach of separating the components into different vectors offers temporal control and safety benefits, delivering multiple components at doses high enough for efficacy without toxicity is a difficult challenge. As is the added complexity of manufacturing and regulation for two different products. However, it is reassuring that success was achieved after a single dose of each vector, perhaps assuaging some of these concerns.

#### 3.1.10. Preclinical Summary

Aided by animal models that can recapitulate the mutation profile and phenotype of disease (although perhaps not yet over a clinically relevant time course) and the recent rise of gene editing tools, impressive steps forward have been made pre-clinically. As results of numerous studies show, the HT1 phenotype has been ameliorated by a range of different vector and payload combinations, with gene editing and integrative approaches showing a clear advantage owing to the ability of corrected hepatocytes to expand in vivo. Data from these in vivo, vectored approaches that correct the *Fah* gene have been summarised below in Table 2. Although initial transduction and editing efficiencies have not been high, the proliferative advantage displayed by the corrective hepatocytes has resulted in phenotype amelioration from as few as ~1 in 10,000 corrected hepatocytes [68]. However, none of these approaches have yet made the leap to the clinic; hence, to assess which approach may be most optimal clinically, similar trials for non-HT1 liver-directed therapy must be studied.

### 3.2. Clinical Results

#### 3.2.1. General

A review of relevant clinical trial data has centred around liver-directed GT trials using similar vector/payload combinations as described in the preclinical FAH literature. In total, twelve liver-directed GT clinical trials using ten different integrative or gene editing systems were identified; one is complete, six have terminated, and four are ongoing.

#### 3.2.2. In Vivo Genome Editing via AAV and Zinc Finger Nucleases in Haemophilia B and Mucopolysaccharidosis (MPS) Type I/II

Three first-in-human studies were identified: MPS type I to deliver the iduronidase enzyme transgene (NCT02702115), MPS II to deliver the iduronate-2-sulfatase enzyme transgene (NCT03041324), and Haemophilia B to deliver the Factor IX enzyme transgene (NCT02695160). Delivery was via a single intravenous administration of AAV6 vectors, including AAV2 ITRs (rAAV2/6), two of which encode the zinc finger nuclease to create double-stranded breaks at a precise target in the albumin locus where a promoter-less transgene is integrated, delivered in a third rAAV2/6 vector. The transgene would then be expressed under the control of the albumin locus. Approximately 13 patients were dosed via a single intravenous infusion across the three clinical trials, and treatment was considered safe and well tolerated with generally minor treatment reactions consistent with other AAV GTs [20,72]. Acute reactions were seen at the highest vector dose of 5 × 10^13^ vg/kg despite prophylaxis. In all studies, enzyme levels from transgene expression were at best transient with no evidence of sustained activity. No on- or off-target indels were identified from 7 patients’ liver biopsies. High patient exclusion rates resulted from pre-existing AAV6 NAbs or the presence of SNPs that could reduce binding affinity and editing efficiency, respectively. The three clinical trials were terminated [73], and the products are no longer listed under the Sangamo pipeline [74].

#### 3.2.3. Ex Vivo GT via Retroviral Transduction of Autologous Hepatocytes and Transplantation for the Treatment of Familial Hypercholesterolaemia (FH)

In 1992, a pilot ex vivo CT was carried out in five patients with FH to address defective low-density lipoprotein (LDL) receptor expression, which necessitates a liver transplant when possible. Preclinical data demonstrated a safe and feasible surgical approach in dogs and non-human primates [75,76]. The study provided definitive data to show GT had a significant effect on one patient and supportive data from two further patients, but the wide variations in disease improvement, without an attributable cause for the variation, and the encouraging data from other in vivo GT systems at the time meant that the development was discontinued [77,78].

It is notable that corrected cells expressing the LDL receptor do not have a proliferative advantage as seen with corrected *FAH^−/−^* cells in preclinical models. Therefore, the gene transfer observed, the uniformly negative immune response, and the potential expression profile observed in this trial could be encouraging data applicable to HT1 GT.

#### 3.2.4. In Vivo GT Using an LNP Vector Packaging Knockout CRISPR-Cas9 mRNA and sgRNA to Treat Transthyretin Amyloidosis or Hereditary Angioedema

##### NTLA-2001

An LNP-CRISPR-Cas9 gene editing system known as NTLA-2001 has shown very promising results in an early CT (NCT04601051). The in vivo, liver-directed GT for the treatment of Transthyretin Amyloidosis, which is a fatal disease resulting from mutation to the *TTR* gene and is characterised by progressive accumulation of misfolded transthyretin (TTR) in different tissues. The function of TTR is to transport thyroxine and vitamin A, but this can be fulfilled by alternative means, and therefore knockout of the *TTR* gene has limited negative effects. Current therapeutics are sub-optimal and do not prevent disease progression. TTR is predominantly manufactured in the liver, making it an ideal GT target. The LNP is designed for IV delivery and liver tropism due to the addition of apolipoprotein, and this encases a 4400 nucleotide CRISPR-Cas9 mRNA plus sgRNA targeted at achieving knockout of the mutated human *TTR* gene [35].

Backed by safety and efficacy in mouse and non-human primate studies (>94% TTR reduction sustained for 12 months), clinical testing commenced in 2020 [79]. Clinical data from NCT04601051 demonstrated durable serum TTR reduction after a single intravenous dose. A total of 55 mg and 80 mg doses were selected after the dose escalation phase, and a total of 62 patients achieved a rapid 91% reduction in serum TTR reduction. A total of 36 patients had reached >12 months follow-up, demonstrating durable TTR reduction of 90% (95% CI 87–93% reduction), and these levels continued in 25 patients, reaching 2 years post-dose. Additionally, clinical outcome measures demonstrated that 92% of patients showed improvement or no change in their NYHA heart function classification. NTLA-2001 was well tolerated across all dose levels [80,81]. A 765 patient randomised, double-blind, placebo-controlled Ph3 study (NCT06128629) using a 55 mg single dose is currently recruiting [82].

##### NTLA-2002

NTLA-2002 is the next CRISPR-Cas9 mRNA plus sgRNA delivered in an LNP in clinical development with a Ph1/2 currently active with completed enrolment (NCT05120830) in patients with hereditary angioedema (HAE), which affects 1/50,000 people and is characterised by severe, debilitating, and life-threatening recurring and unpredictable inflammatory attacks in various organs of the body [83]. Again, the LNP targets the liver, but in this indication, the sgRNA targets deactivation via knockout of the *kallikrein B1* gene, which leads to reduced kallikrein activity and prevents attacks in HAE patients.

Ph1/2 data demonstrates NTLA-2002 was well tolerated across all dose levels, with no grade 3 or above AEs and no SAEs. Monthly attack rates reduced by 80% and 81% during weeks 5–16 in the 25 mg and 50 mg single-dose arms, respectively, compared to placebo. At 8 months, complete response with no attacks was observed in 8 of 11 patients in the 50 mg arm, compared to 4 of 10 in the 25 mg arm and 0 in the placebo arm. The 50 mg arm achieved kallikrein protein reduction of 86% from baseline compared to 55% in the 25 mg arm at week 16 [84]. The phase 3 (NCT06634420) study is currently recruiting [85].

Although these two clinical trials are investigating gene knockout, rather than editing, the potential to deliver CRISPR-Cas9 mRNA product to the liver in a liver tropic LNP, which could be a safe and effective way of transducing hepatocytes with gene editing product for HT1.

#### 3.2.5. In Vivo LNP Delivery of an Adenine Base Editor (ABE) to Treat FH, Atherosclerotic Cardiovascular Disease and Uncontrolled Hypercholesterolaemia

VERVE-101 is currently being tested in a Ph1 trial and is given via a single intravenous infusion (NCT05398029). The liver-targeted LNP package is the ABE mRNA plus an sgRNA to induce a point mutation in the *PCSK9* gene, which subsequently lowers LDL in the blood. Dose escalation cohort data from the first 10 pts dosed at one of four dose levels (0.1 mg/k; n = 3, 0.3 mg/k; n = 3, 0.45 mg/k; n = 3, and 0.6 mg/k; n = 1) demonstrated significant reductions in PCSK9 (47%, 59%, and 84%) and LDL-C (39%, 48%, and 55%) in three of the four participants in the 0.45 mg/kg and 0.6 mg/kg cohorts, which were sustained over the observation period of up to 180 days. Two grade 3 or above treatment-related AEs were observed as well as raised, transient ALT levels of up to 300 U/L [86]. Recruitment into the two higher dose levels continued, but the study was put on dosing hold in Apr 2024 following additional treatment-related SAEs [87]. Verve Therapeutics are now prioritising the development of VERVE-102 in FH patients, with a Ph1b clinical trial recruiting (NCT06164730). VERVE-102 uses the same base editor and guide RNA for *PCSK9* but a modified lipid nanoparticle (LNP) to deliver the cargo [88].

The use of an LNP vector to deliver cargo to the liver and the ability to change single nucleotides could be translatable to the correction of *FAH^−/−^* hepatocytes. Preclinical LNP/BE HT1 studies were reported by Song et al. and Jiang et al. in mice where editing efficiency was reported up to 12.5% before expansion, using multiple high LNP doses.

#### 3.2.6. In Vivo AAV Vectors Carrying Human Enzyme Genes Plus Homology Arms for Integration into a Specific Locus

##### hLB-001

Methylmalonic Acidemia (MMA) is a severe, sometimes lethal, monogenic inborn error of metabolism with onset during infancy caused by a mutation in the *human methylmalonyl-CoA mutase (MMUT)* gene resulting in high methylmalonic acid in all body fluids, causing metabolic crisis. It is unsatisfactorily managed with a low-protein diet and requires a liver transplant to stabilise metabolic activity [89]. “GeneRide” technology, hLB-001, is a recombinant AAV vector utilising the LK03 capsid, designed to non-disruptively integrate the *MMUT* gene at the albumin locus via HDR. After promising results in mice, including corrected MMUT+ hepatocyte expansion despite initial editing rates of <1% [90], a paediatric Ph1 CT (NCT04581785) was initiated. The study was placed on hold in 2022 due to patients developing thrombotic microangiopathy and then terminated due to low likelihood of clinical benefit in treated participants [91,92]. Basic results are available on https://clinicaltrials.gov/ showing that although some efficacy parameters demonstrated changes, the safety profile was not favourable [91].

##### HMI-103: AAVHSC15 Plus *Human Phenylalanine Hydroxylase* Gene cDNA with Homology Arms for Integration into the PAH Locus to Treat PKU

A Ph1 adult CT (NCT05222178) was designed to test HMI-103, an AAVHSC15 vector packaging the human *PAH* gene cDNA plus homology arms designed to integrate into the *PAH* locus via HDR and restore PAH activity and normalise Phenylalanine metabolism. PKU is a rare autosomal-recessive inborn error of metabolism that can result in progressive, irreversible neurological impairment. HMI-103 has the potential to produce the PAH enzyme via genome integration as well as episomal expression to restore phenylalanine metabolism. A single IV delivery of HMI-103 plus prophylactic immunosuppression was used to ameliorate T-cell responses against AAV transduced cells and reduce anti-AAV antibodies [93]. In July 2023, initial results from the first dose level were announced on the company website. Three patients had been dosed, and HMI-103 had been generally well tolerated with no SAEs or abnormal LFTs. Participant 1 “achieved clinically meaningful reduction in plasma Phe of up to 99% from baseline with most levels below the treatment guideline threshold (<360 μmol/L) until 31 W post-dose. Patient 2 achieved plasma Phe reduction of 49% at 17 weeks from baseline but had not achieved levels <360 μmol/L, potentially due to self-liberalised dietary protein. Patient 3 had recently been dosed [94]. Later the same day a further announcement was made: “…based on the current financing environment and Homology’s anticipated clinical development timelines, Homology will not be further developing its programs…while it explores options for the Company and its assets, including HMI-103” [94]. The study was terminated in October 2023.

## 4. Discussion

An abundance of preclinical data are available demonstrating successful amelioration of the HT1 phenotype in *FAH^−/−^* animal models. Mouse, rat, rabbit, and pig models all demonstrate that corrected FAH+ cells have a strong selective proliferation advantage in the mutant liver and can expand through normal growth or when hepatocyte injury is induced by withdrawing the protective effect of NTBC. Many different vector and payload combinations have been used to demonstrate this phenomenon with combinations that allow integration of corrected alleles either via gene editing or insertion of an *FAH* transgene, resulting in long-term FAH expression and disease cure. Why, therefore, have no HT1 GT clinical trials commenced?

### 4.1. After Review of the Available HT1 Animal Model Data and Relevant Liver-Directed Clinical Information, Would a Single-Dose GT Approach Be Viable to Cure HT1 Considering the Scientific, Economic, and Ethical Landscape?

#### 4.1.1. Scientific Factors

The phenotypic cure of HT1 is demonstrated by normalisation of biochemical parameters and the production of sufficient FAH enzyme to allow the metabolism of tyrosine to proceed through all five steps. This results in the absence of toxic metabolites and associated liver injury manifestations and allows normal animal growth. This was observed in several preclinical studies. Correction of just one allele per hepatocyte is enough to allow the cell to express FAH and inhibit the accumulation of FAA, MAA, and SA. In genetic diseases where the liver does not suffer predominant pathological injury, such as Haemophilia A and B and Fabry disease, threshold amounts of enzyme expression may suffice to cure disease phenotype without requiring corrected cell expansion. However, in HT1, cells that remain *FAH^−/−^* would not be protected upon NTBC withdrawal and would continue to accumulate toxic metabolites, leading to liver fibrosis, cirrhosis, and potentially HCC, as demonstrated in several preclinical studies in mice and pigs using different vectors [58,59,95]. Therefore, GTs curative potential in HT1 is reliant on transgene expression levels and on the proliferative potential and fate of FAH+ cells in the short and long term. Notably, almost complete FAH+ hepatocyte repopulation was realised in several longer-term studies, but this took time and was not necessarily dependent on high initial editing efficiency. For example, the fastest time (49 days) to >90% repopulation was achieved having provided GT in utero in mice using 2xAAVs-Cas9-sgRNA-HT [28]. The relative timing of GT dosing and NTBC withdrawal will be important to optimise. When considering translation into humans, a liver trophic vector/payload that transduces cells at higher frequencies should be considered to avoid variable outcomes. The higher the initial editing efficiency is, the more nodules are created, which can proliferate.

After repopulation with FAH+ cells, the rate of mitosis, in both mutant and corrected cells, reduces greatly, decreasing the likelihood of the development of HCC from Fah- cells [54]. Immune tolerance, integration profiles, indels, and off-target activity are all concerning when considering the scientific feasibility of developing a curative GT. However, favourable safety outputs relating to all these metrics have been shown.

The proliferative potential of corrected hepatocytes in the *FAH^−/−^* environment and the many examples of different vector/payload combinations being well tolerated provide evidence that GT for HT1 would be viable and safe from a scientific perspective.

#### 4.1.2. Economic Factors

The cost of NTBC treatment in the US is incredibly high, with total HT1 management costs likely to be over $1 M/year/patient. Even with the high price of GTs often over $1.5 M [96], in comparison to current spending on HT1, this would represent good value, even in the UK where NTBC costs are much lower. However, high upfront costs to payers for therapies where long-term outcomes are unknown are difficult to justify. This is exemplified by the fate of Glybera, a promising GT for Lipoprotein Lipase Deficiency which was approved in the EU in 2014 and then withdrawn in 2017 due to the unwillingness of payers to pay [12], and the Marketing Authorisation has now expired [97]. This makes investment in potential one-shot curative therapies an unattractive prospect when long-term continual therapies might be less risky for economic forecasts [98]. Pricing models for new therapies should be based on R&D costs, the number of potential recipient patients during the period of patent protection, and the manufacturing costs and profit margin, with payers, considering the comparison to the current standard of care and improvements to QALYs [99]. GT manufacturing costs are high, especially those using hard-to-produce clinical-grade vectors such as Lentiviruses, estimated at $300,000/vector dose [100], or expensive ex vivo processes. R&D costs are also high and hard to predict in this new field, with complex clinical trials, hard-to-recruit patients, and long-term follow-ups required, as well as CMC and regulatory requirements. Such pressures seemed to drive the discontinuation of HMI-103 development and likely many other GT development programs rather than scientific failure. Patient uptake of new therapies is hard to predict, especially when NTBC is available as a known entity with tolerable outcomes. Uncertainty around these important price-determining factors combines to make GTs such as Lidmeldy the most expensive of any therapeutic at £2.8 M [98].

For a company to take a risk on developing a GT for HT1, a few advantages may require emphasising. Firstly, the current question over the longevity of episomal AAV expression is not relevant in the HT1 context, as pre-clinical data suggests a successful delivery and repopulation of corrected hepatocytes is more likely to achieve a true one-shot fix with all other management costs reduced to occasional follow-up. Additionally, with onset in infancy and the advantage of a growing liver inclined to using HDR correction mechanisms, patient uptake could eventually be reliable and existing vector immunity concerns either irrelevant or very low.

With the high prevalence of GT clinical trials and licensure gaining momentum, with a predicted 10–20 gene and cell therapy approvals per year by 2025 (FDA.com, 2020), there is a need for sustainable pricing models to ensure that the development of one-shot cures remains an attractive goal for both payers and industry. Kerpel-Fronius et al. suggests a model where transparent sharing of costs and financial risks, including the burden of post-authorisation regulatory commitments, is considered for a risk-sharing program between industry society and governments, and long-term payment plans are available with caveats for treatments that do not endure [99]. Fortunately, complex manufacturing associated with GTs is being addressed by the investment in automation and pod factories, which should ultimately reduce the risk and cost of manufacturing long-term (fiercepharma.com, 2021). LNP/mRNA COVID-19 vaccine technology has resulted in efficient and enhanced manufacturing processes and manufacturing cost reductions. This trend towards production cost reduction coupled with approaches to enhance in vivo delivery and thereby dose-sparing will be important for future viability.

Although the current economic climate for GT developments is challenging, future health benefit realisation and financial incentives may improve economic viability.

#### 4.1.3. Ethical Considerations

Several ethical questions need to be considered to ensure clinical trials have a suitable benefit/risk profile for HT1 patient participation. As is consistently seen in the preclinical data, corrected cell expansion needs to occur, and this repopulation takes time and is triggered when liver injury is induced by NTBC withdrawal, but this leaves cells unprotected. As corrected cells expand, apoptosis occurs in uncorrected cells, and areas of the liver are at risk of developing fibrosis, cirrhosis, and possibly HCC [2]. Liver biopsies in pigs 225 days post-treatment with LV-FAH therapy revealed that while the liver was still repopulating with corrected cells, other liver regions contained multiple abnormalities and fibrotic patches, which then resolved to WT levels by day 337 [58]. Cycling NTBC on and off following dosing has been used in many studies to induce liver injury and correct cell expansion while rescuing the animals from weight loss and preventing other HT1 acute symptoms like metabolic crisis. The ethical question of how to manage these risks in a CT is important, as exposing humans to the unknown, unquantifiable risk of developing irreversible liver damage, HCC, or a metabolic crisis is unjustifiable. In order to manage this risk, close follow-up would be required to ensure metabolic and biochemical parameters remain acceptable as well as appropriate rescue mediation guidance (i.e., NTBC dosing). Some AAV studies using Cas9 products compared the initial editing efficiency of leaving animals on NTBC post-dosing for a short or longer period. A base editor in 2xAAVs was delivered, and a 3.8% vs. >11% editing efficiency was observed with mice on NTBC for 7 days post-dosing vs. 21 days [66]. The LV-FAH studies all withdrew NTBC at or shortly after dosing and then required several rounds of NTBC cycling, which may be impossible in humans. NTBC continuation for a period after dosing or perhaps reducing the dose of NTBC gradually may be a less risky strategy in adult humans.

The clinical trial population should be well defined. For maximum benefit, GT would be best employed in infants, and the power of the neonatal liver to repopulate quickly and expand corrected cells supports this [64,69,90]. However, as treatment currently exists, clinical trials should start in adults who have poor control. A challenge exists in defining exclusion criteria, though, as poorly controlled HT1 human adults may have liver disease involvement, which is usually an exclusion criterion in liver-directed GT trials (NCT05487599; NCT04370054; NCT02484092; NCT04040049). Thorough toxicology studies would be needed, like that performed using LV-FAH [57]. Progressing clinical trials to neonatal (or in utero) populations presents additional ethical challenges, for example, parental consent and long-term safety uncertainties, and would warrant careful consideration, especially given the existing availability of a low-risk therapeutic.

Rossidis et al. demonstrated high gene editing potential in mouse foetuses using the *Hpd* gene knockout method [60], and Mondal et al. also confirmed in utero editing efficiency of the *FAH* gene; however, in utero dosing is associated with extensive ethical questions and, at present, would be better avoided. Notably, the study also found that 14-day-old neonatal hepatocytes possessed the highest amount of HDR repair, genomic correction, and *FAH* gene expression [28]. As neonates have such high gene correction potential and the growing liver will expand corrected hepatocytes, if growth patterns match those in humans, this would appear to be the ideal time to dose HT1 patients.

### 4.2. Which Vector and Payload Combination Would Be Most Suitable to Pursue into Clinical Development?

Many different vector and payload combinations have demonstrated transduction of hepatocytes in *FAH^−/−^* animal models, which have eventually led to phenotype amelioration, but some combinations have distinct advantages over others, which should be considered to maximise success in the clinic. Choosing a vector and payload combination that has reliable initial transduction efficiency with safe integration and off-target activity profiles to create enough corrected, healthy, and stable cells that can repopulate the liver once NTBC is withdrawn is important.

#### 4.2.1. Vectors

##### Lentiviral Vectors

The most comprehensive and long-term large animal data supportive of clinical progression come from the LV-FAH vector/payload combination with almost complete repopulation of the liver with corrected hepatocytes after 9–12 months and complete amelioration of the HT1 phenotype. The integration profile, which, although prolific and widespread, appears benign with a shift over time favouring proliferation of cells with integration sites in introns rather than exons and in non-tumour-associated genes [58]. In vivo, efficacy and safety data are thorough and impressive and support further development in the clinic. Notably, no clinical trials have been performed using liver-directed in vivo administration of LV. Anti-vector responses are one barrier to translatability [58], which needs to be addressed as they currently drive reliance on dosing via ultrasound-guided, percutaneous portal vein infusion.

##### Adeno-Associated Viral Vectors

Whilst current FDA-approved haemophilia GT relies on AAVs natural episomal maintenance, this approach is not ideal for HT1; instead, editing tools such as CRISPR-Cas have been relied upon. Several reports of successful dose-dependent integration, high editing efficiency, and corrected hepatocyte expansion can be found in the preclinical HT1 literature [28,66,90] using AAV or AAV and LNP combined systems [71]. Maintaining mice on NTBC for longer periods after dosing also appeared to increase initial editing rates [66,90]. Notably, all these studies were performed in mice rather than pigs. Studies in larger animals or in mice with chimeric livers with human hepatocytes would be helpful to gain further insight [101].

Numerous liver-directed clinical trials and licensed therapies exist using AAVs, which are generally safe and well tolerated; however, AAVs can trigger both innate and acquired immune responses [89]. Loss of transgene expression has been associated with dose-dependent T-cell responses, which may be stabilised by corticosteroid use [102]. Anti-AAV acquired immunity prevalence in the general adult population is generally around 50% depending on serotype, age, and geography [103], thus reducing the suitable patient pool, which is problematic in such a rare disease. The use of recombinant AAVs or dosing in neonates may be viable to increase the number of patients that could benefit.

Indel frequency was high for Cas9 systems [62,69]; off-target activity was variable but generally did not demonstrate significantly higher frequencies than found in WT animals even using DNA rather than mRNA to encode Cas9 [28,64,71,95]. Additional analysis regarding the safety of indels created via Cas9 and NHEJ would be helpful.

Editing specific mutations is a personalised medicine strategy where each mutation would require a specifically designed and tested product, the economic modelling for which is far more restrictive than inserting a “one size fits all” complete transgene. Even though liver-directed BEs and Cas9 products are in use in the clinic for other diseases, they knock out genes rather than correcting them, which is a) easier to achieve and b) does not require a personalised strategy and is, therefore, economically viable. The requirement for dual AAV systems for delivery may also limit efficacy because dosing and cost could be restrictive.

The addition of Cas9-sgRNA or Cas9n-sgRNA could potentially increase the initial editing efficiency as per the data from the Di Giorgio study, which demonstrated guided integration via a dual AAV system delivering saCas9-sgRNA plus *FAH* transgene with Apoa1 sequence homology arms [65]. This was a small and short study of only 40 days, but the Cas9-sgRNA significantly increased initial editing.

##### LNPs

Preclinical data in HT1 animal models using LNPs is limited but potentially encouraging. A BE with a 0.44% initial editing efficiency was delivered [51], and an LNP was used to deliver Cas9 mRNA with an AAV to deliver sg-RNA and a repair template, achieving an impressive >6% initial editing efficiency in adult mice [71].

Additionally, three of the liver-directed gene editing products with ongoing clinical trials use LNPs to deliver Cas9 products, demonstrating high editing potential in humans (NCT05398029, NCT05120830, NCT04601051) and correction of disease biomarkers in a high proportion of patients. LNP technology presents an exciting option for delivering GT with fewer immune system challenges, increased adaptability, and manufacturing and scalability cost benefits in comparison to their viral vector counterparts; however, data in HT1 models is limited.

### 4.3. Summary

As treatment exists for HT1 patients, a GT alternative must provide significant therapeutic advantages over NTBC. When choosing the optimum vector/payload GT combination, the priority should be to find the safest and most broadly applicable to all HT1 patients, ideally from birth. The LV-FAH vector/payload combination is the only system that has demonstrated extensive, long-term, and reproducible positive results in a large animal and, therefore, the only system that would likely get to clinic in the short term. However, an in vivo liver-directed LV clinical trial would be novel and need careful planning to address risks.

LNPs present an attractive vector that can be optimised for liver tropism and can deliver large cargos without eliciting concerning immune responses. Clinical data in other genetic indications is being generated at present, which may be translatable to future HT1 GT. AAVs are also an attractive choice, especially given their wide use already in liver-directed GT, but innate and acquired immune responses may reduce broad applicability.

Although it is possible to correct specific mutations using various next-generation CRISPR products, it may be a long while before personalised GT systems become economically viable, and therefore a payload involving a full *FAH* gene insertion is a more realistic option at present. Unfortunately, two clinical trials using AAVs to deliver an FAH transgene with homology arms in the absence of Cas9 were discontinued in 2023 (NCT05222178 and NCT04581785) due to economic and potential efficacy consistency reasons, which highlights the challenging landscape for GT development at present.

Ideally, further long-term, large animal studies would be carried out to investigate the potential of LNP or AAV vectors with targeted insertion of full *FAH* transgenes with or without CRISPR tools.

### 4.4. Limitations

The main limitation of this study is that although there is an abundance of preclinical data comparing different vector/payload combinations, most studies were proof-of-concept using the useful *FAH^−/−^* model to test the viability of GT systems to make stable changes to hepatocyte genomes rather than focusing on curing HT1. The LV-FAH system is much more advanced than all other systems but is not necessarily the most ideal. Human clinical data of integrational liver-directed GTs is immature at present and a few years away from being truly useful to inform which vector/payload combination might be the safest and most reliably effective in HT1.

In terms of model validity, there are dramatic differences in the scale of human vs. mouse livers, and the size of fenestrated endothelia in the liver is different (~100 nm in humans, 130–160 nm in mice [16]), impacting the clearance of particles into the liver. Furthermore, most hepatocytes are polyploid in adult rodents. Polyploidy increases with age and during liver injury, such as partial hepatectomy or oxidative stress [104], and the rate varies between animals, with mean hepatocyte ploidy being >5 in mice, ~3.18 in pigs, and ~2.15 in humans [105], which may affect the reproducibility of data in humans, as the percent allele correction required would be higher with lower polyploid cells. However, polyploidy is also associated with a higher likelihood of carcinogenesis [106], which may be more favourable in humans with lower rates of polyploidy. It is also notable that there are anatomical differences between pig and human livers, which may reduce their validity as a model despite their more appropriate scale.

## 5. Conclusions

GT has the scientific potential to provide a single-dose cure for HT1 patients, supported by potential economic viability and an unmet medical need. A significant amount of preclinical data has been generated using four animal models of HT1 and a range of vector/payload combinations and has demonstrated that, even with low initial editing efficiency, the *FAH^−/−^* liver environment provides a selective proliferation advantage where corrected cells will repopulate the liver. For this reason, an integrative or genome editing vector/payload combination should be chosen to move forward into clinical development; this may take the form of lentiviral or CRISPR/LNP approaches. At present, the most abundant data supporting a move into clinical development is using an LV-*FAH* integrative transgene approach with multiple studies in pigs demonstrating success ameliorating the HT1 phenotype and providing long-term, stable integration and expression. Many other vector/payload combinations have resulted in this same success but in smaller animals. Integrative or edited liver-directed GT clinical data are currently limited, but several ongoing clinical trials are likely to provide important relevant data over the next few years. Finding the safest, most reliable vector/payload combination for application in neonates as well as later in life would be the ideal way to gain maximum benefit from a curative HT1 GT.

## Figures and Tables

**Figure A1:**
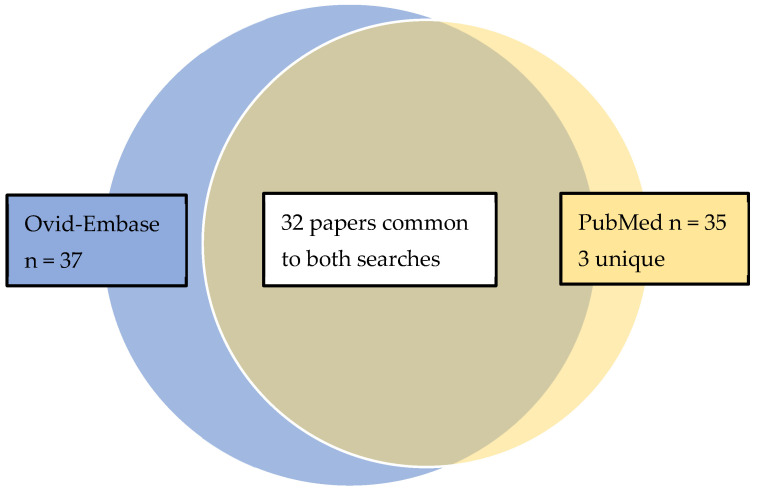
Venn diagram showing papers overlapping in PubMed and Ovid-Embase searches.

## Appendix A

Preclinical Search
Data bases and search criteria

A comprehensive literature search was performed using the electronic databases PubMed [37] and, as a validation step, Ovid-Embase [38] to retrieve English language papers (which may introduce bias but was necessitated by the available time and resources). The last search date was 15 November 2024.

Publications reporting pre-clinical studies with results investigating the use of GT systems in animal models of Hereditary Tyrosinemia Type 1 were of interest. These were identified using the following criteria.

PubMed Search:

((“Genetic Therapy”[Mesh]) OR (“CRISPR”[Text Word] OR “nucleotide therap*”[Text Word] OR “Gene Delivery”[Text Word] OR “gene edit*”[Text Word] OR “genetic edit*” [Text Word] OR “genetic therap*”[Text Word] OR “gene therap*”[Text Word] OR “gene targeted therap*”[Text Word] OR “DNA therap*”[Text Word])) AND ((“Tyrosinemias”[Mesh]) OR (“Tyrosinemia Type 1”[Text Word] OR ht1[Text Word]))

64 results were found.
Ovid-Embase Search:

(exp GT/or (CRISPR or “nucleotide therap*” or “gene edit*” or “genetic edit*” or “gene delivery” or “genetic therap*” or “gene therap*” or “gene targeted therap*” or “DNA therap*”).ti,ab,kw) AND (tyrosinemia/and or (tyrosinemia* or ht1).ti,ab,kw).

235 results were found.
Preclinical study exclusions:

No other filters were applied for either search. Both searches were manually reviewed, and the following exclusion criteria were applied.

**Table A1:** Manual review and exclusions of PubMed and Ovid database searches.

Exclusion Criteria	Rationale	PubMed “Hits” Removed	Ovid “Hits” Removed
Review article, editorial, short survey, or HT1 general publications (focused on treatments, new-born screening, progress etc.)	General reviews not focused on HT1 preclinical study outcomes. Opinion/Potential bias in interpretation	17	119
Pre-print, conference abstract or papers, letters	Not peer-reviewed. Data often available in other papers	0	55
Duplicate PC study hits		0	8
Different Indication (e.g., cancer, HT3)	Does not align with focus of this dissertation	1	1
Generation of an HT1 animal model	Not a GT study, not the focus of this dissertation	4	5
Cell therapy study	Does not involve GT, not the focus of this dissertation	3	2
mRNA multi-dose study	Not a single treatment cure, not the focus of this dissertation	1	2
Other publications not focused on GT HT1 treatment (e.g., lipid or oral medication study)	Does not align with focus of this dissertation	1	2
Liver transplant publication	Not GT, not the focus of this dissertation	1	1
Not a peer-reviewed article	Peer review is a metric of quality	0	0
GT method experiment but without any HT1 quantitative or phenotype data	No useful data from which to draw conclusions	0	1
Surgical or cell culture method for ex vivo system	Supplementary information—No outcomes reported	1	2
**Total exclusions**		**29**	**198**
**Total publications included**		**35**	**37**
**Total publications in search**		**64**	**235**

Only pre-clinical studies that aimed to correct the mutation and phenotype of HT1 via GT were included in the analysis. Of 64 results from PubMed and 235 from Ovid-Embase, a total of 40 relevant publications remained, which described 39 relevant preclinical studies, as one paper contained long-term data from a previous study. Approximately 32 of these publications appeared in both the PubMed and Ovid-Embase searches.

Final Search Output:

A total of 40 papers with publication dates between 1996 and 2023 were reviewed and included in the pre-clinical GT for the HT1 review. This review provided the primary information to answer the research questions of this dissertation.

Clinical Search:

The second step involved a review of relevant translatable clinical data. A search of clinicaltrials.gov, Pubmed, and SOLO databases failed to identify reported GT clinical trials for HT1.

As an alternative, data from other liver-directed GT clinical trials were used to try and assess the translatability of the preclinical approaches so far reported. After consideration of the preclinical data and HT1 disease pathology literature, the primary focus was to search for clinical trials that involved GT systems that either integrate into the genome or edit the genome itself.

For this search, clinicaltrials.gov [107] was used with the ‘search term’ “Gene Therapy” and ‘search details’ used to restrict this to “Genetic Diseases”. On 5 May 2023, 807 initial records were found. This large number results from clinicaltrials.gov searching ‘gene’ and ‘therapy’ as separate terms as well as together. These were downloaded to an Excel file for easier filtering. Exclusions included: 89 cancer trials, 391 trials because they were not interventional GT trials, and 251 trials were not liver directed, for example, those targeting the eye via intraretinal delivery or those delivered intra-cranially, targeting the CNS or via haematopoietic stem cell transplantation. This left 76 liver-directed GT trials. The final step was to review mechanisms and to sort them into those that used AAVs encoding genes to be primarily maintained episomally and those that used an editing or integrative approach. In total, twelve liver-directed GT clinical trials using ten different integrative or gene editing systems were identified; one is complete, six have terminated, and four are ongoing.
